# Investigation of early and lifetime clinical features and comorbidities for the risk of developing treatment-resistant depression in a 13-year nationwide cohort study

**DOI:** 10.1186/s12888-020-02935-z

**Published:** 2020-11-17

**Authors:** Shiau-Shian Huang, Hsi-Han Chen, Jui Wang, Wei J. Chen, Hsi-Chung Chen, Po-Hsiu Kuo

**Affiliations:** 1 Department of Public Health & Institute of Epidemiology and Preventive Medicine, National Taiwan University, Taipei, Taiwan; 2 Bali Psychiatric Center, Ministry of Health and Welfare, New Taipei City, Taiwan; 3 Department of Psychiatry, College of Medicine, National Yang-Ming University, Taipei, Taiwan; 4 Taipei Veterans General Hospital, Taipei, Taiwan; 5 Department of Psychiatry, Yang Ji Mental Hospital, Keelung, Taiwan; 6 Department of Psychiatry, National Taiwan University Hospital, Taipei, Taiwan; 7 National Health Research Institutes, Center for Neuropsychiatric Research, Miaoli County, Taiwan

**Keywords:** Treatment-resistant depression, Antidepressants, Early and lifetime comorbidities, Prescription pattern, Health-seeking behavior

## Abstract

**Background:**

To investigate the risk of treatment-resistant depression (TRD) in patients with depression by examining their clinical features, early prescription patterns, and early and lifetime comorbidities.

**Methods:**

In total, 31,422 depressive inpatients were followed-up from diagnostic onset for more than 10-years. Patients were diagnosed with TRD if their antidepressant treatment regimen was altered ≥two times or if they were admitted after at least two different antidepressant treatments. Multiple Cox regression model were used to determine whether physical and psychiatric comorbidities, psychosis, and prescription patterns increased the risk of TRD by controlling for relevant demographic covariates. Survival analyses were performed for important TRD-associated clinical variables.

**Results:**

Females with depression (21.24%) were more likely to suffer from TRD than males (14.02%). Early anxiety disorders were more commonly observed in the TRD group than in the non-TRD group (81.48 vs. 58.96%, *p* < 0.0001). Lifetime anxiety disorders had the highest population attributable fraction (42.87%). Seventy percent of patients with multiple psychiatric comorbidities developed TRD during follow-up. Cox regression analysis further identified that functional gastrointestinal disorders significantly increased TRD risk (aHR = 1.19). Higher doses of antidepressants and benzodiazepines and Z drugs in the early course of major depressive disorder increased TRD risk (*p* < 0.0001).

**Conclusion:**

Our findings indicate the need to monitor early comorbidities and polypharmacy patterns in patients with depression associated with elevated TRD risk.

## Background

Major depressive disorder (MDD) is a common mental disorder with high lifetime prevalence [1]. The average age of onset for MDD ranges from the patient’s early twenties to their late thirties [1]. The symptoms of MDD significantly impair patients’ daily functions and a substantial proportion of patients with MDD do not respond to standard antidepressant treatment regimens [1]. Patients who do not respond to antidepressants are likely to suffer from treatment-resistant depression (TRD) [2]. Treatment resistance increases medical burden and individual and societal costs [3] The systematic evaluation of TRD and understanding its risk profiles, disease mechanism, prescription patterns, and the impacts on the mental health system may help in developing strategies to prevent the adverse consequences associated with TRD.

The criteria for TRD has been defined by European Medicines Agency as the failure to benefit from at least two different antidepressant treatments with sufficient duration at an adequate dose [4–7]. The proportion of patients with TRD among those with MDD varies between studies (ranging from 6 to 50% according to data-based analyses vs. clinical studies) due to differences in study designs and definitions of TRD [8]. Patients with TRD tend to have more frequent hospitalization, recurrent depressive episodes, and emergency room (ER) visits [2, 6, 9]. In addition, outpatient care is insufficient for a substantial proportion of patients with MDD despite advances in clinical care, treatment regimens, and drug development. Approximately 8.3% of patients with MDD are hospitalized annually [10]. These patients often exhibit worse symptoms, increased comorbidities, and higher suicide risk, and are prescribed higher doses of antidepressant [11]. MDD patients who were ever admitted to the psychiatric ward represent a prominent subgroup requiring intense care and complex treatment regimens; they are at a high risk of developing TRD.

Several clinical and psychosocial factors have been reported to be associated with poor treatment response in patients with MDD, including poor treatment adherence, physical and psychiatric comorbidities, bipolarity, and high level of neuroticism [6, 12–14]. Physical comorbidities, including diabetes [15, 16], heart diseases [17], functional gastrointestinal disorders (FGIDs) [18], thyroid disease [19], systemic lupus erythematosus (SLE) [20], and rheumatoid arthritis [21], have been implicated in the development and prognosis of common mental disorders, especially mood disorders. In addition, a group of anxiety disorders (including panic disorder, generalized anxiety disorder, social anxiety disorder, and obsessive-compulsive disorder), attention deficit hyperactivity disorder (ADHD), substance use disorders (SUDs), and personality disorders were found to be more prevalent in patients with MDD [22–25], or predictive of worse outcomes or slower remission [24]. This emergent evidence has indicated that both physical and psychiatric comorbidities might prolong the course of depression and worsen clinical outcomes. However, the magnitude of impacts of comorbidities in disease onset or during disease course on treatment resistance in depression are not well studied for MDD. For the former, a prospective study design is required to evaluate effects of early comorbidity status on the risk of developing TRD.

Patients taking antidepressants often have poor adherence, resulting in inadequate treatment effects and a high risk of developing TRD [8, 14, 26]. In clinical settings, it would be helpful to recognize the patterns of health-seeking behavior and medical prescriptions during the onset or early in the course of MDD to improve the management of TRD patients. We used a large-scale claims-based database covering the majority of psychiatric patients ever admitted to a hospital to minimize selection bias and compared the results with those of ordinary observational studies or clinical trials. In this database, MDD patients were followed-up from the onset of diagnosis until year 2011. We quantified the proportion of TRD in the nationwide database, evaluated the general characteristics of TRD, and investigated the risk profiles. We examined the effects of physical and psychiatric comorbidities in lifetime, occurring at the onset, or during the course of MDD on the risk of developing TRD. There were several goals in the present study. First, we used a retrospective study design to evaluate feature of TRD in lifetime. Second, we aimed to evaluate the influence of early prescription patterns, health-seeking behavior, and the severity of depressive symptoms during the first year of MDD diagnosis in patients who developed TRD versus those who did not develop TRD during follow-up. Third, using a prospective study design and analytical approaches, the influence of comorbidities on the risk of TRD was investigated using the time-to-event (TRD occurring) method during 13-years of follow-up in patients with MDD. Lastly, the population attributable fraction (PAF) was calculated to estimate the impact of important comorbidities associated with TRD.

## Methods

### Data source and study population

**Fig. 1:**
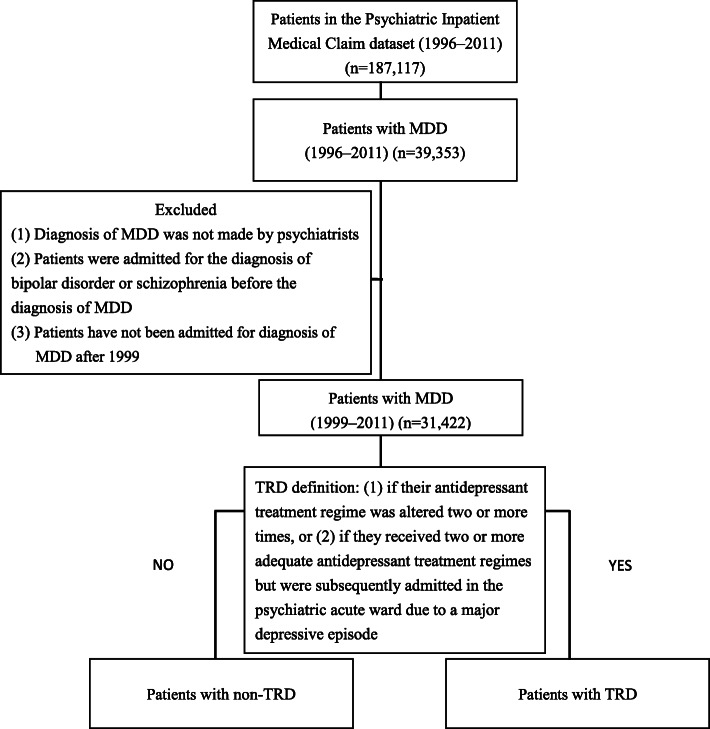
Consort diagram of participants’ selection. Abbreviations: TRD, treatment-resistant depression; MDD, major depressive disorder

The national health insurance program was launched in 1995 to finance healthcare for all individuals in Taiwan and covered approximately 97% of the population. The national health insurance research database contains the Psychiatric Inpatients Medical Claim (PIMC) dataset, which includes all patients who were ever hospitalized for any psychiatric diagnosis (*n* = 187,117). The PIMC dataset includes basic demographic information and comprehensive medical records (e.g., medication, outpatient service, and emergency visit) [27]. Individuals included in the PIMC dataset with a principal inpatient diagnosis of MDD (ICD-9-CM code 296.2–3, *n* = 39,353) between 1996 and 2011 were eligible for inclusion in the present study (Fig. 1). We targeted patients who had ever been admitted to a hospital because of depressive episodes. Patients were excluded if (1) MDD diagnosis was not made by a psychiatrist, (2) if they were diagnosed with schizophrenia (ICD-9-CM code 295) or bipolar disorder (BpD) (ICD-9-CM code 296.0, 296.1, and 296.4–8) before MDD diagnosis, or (3) patients have not been admitted for diagnosis of MDD after 1999. All medical records, and diagnoses of physical and psychiatric comorbidities based on ICD-9-CM codes were then obtained for MDD patients during the whole study period. Taken together, 31,422 patients with MDD were included in the present study. The longest follow-up duration was 13 years.

### Operational definition of TRD

Depression is usually classified as TRD when at least two treatments of different antidepressants (adequate in terms of dosage and duration) fail to produce a significant clinical improvement. In the present study, we defined patients as treatment-resistant (1) if their antidepressant treatment regimen was altered two or more times for different antidepressants, or (2) if they received two or more different adequate antidepressant treatment regimens but were subsequently admitted to the psychiatric acute ward due to a major depressive episode [4, 7]. An adequate antidepressant trial was defined as having greater than 56 cumulative defined daily doses (DDD) for each antidepressant treatment over at least 8 consecutive weeks [6, 28].

### Independent variables and covariates

Demographic and clinical data between TRD and non-TRD groups were extracted from PIMC. The demographic features were age, sex, urbanization level, and income (estimated via insurance). The urbanization level of patients was stratified as seven levels, with level I being the most urbanized and level VII being the least urbanized [29]. Clinical information included psychiatric and physical comorbidities, prescription patterns, records of medical utility, all-cause mortality, conversion rate to another diagnosis during follow-up, and time from the MDD diagnosis to another diagnosis. Data of physical comorbidities were collected: diabetes, SLE, rheumatoid arthritis, cardiovascular disease, renal disease, FGIDs, and thyroid dysfunction. The psychiatric comorbidities were personality disorders, ADHD, SUDs, anxiety disorders, panic disorders, and non-organic psychosis. In terms of medical utility, we collected information on the number of ER visits, admissions to all specialized departments, and outpatient visits for patients diagnosed with MDD. During the long-term follow-up, a patient with an original diagnosis of MDD may be diagnosed with other disorders, such as BpD and schizophrenia [12], which might contribute to difficulties in original treatment using antidepressants; therefore, the conversion rate to BpD or schizophrenia was calculated. The all-cause mortality rate was also evaluated to indicate the long-term prognosis of patients with or without TRD development.

All comorbidities in the claims database were defined by at least three outpatient visits or one inpatient care visit that was linked to the diagnosis. Early psychiatric and physical comorbidities were defined as follows, those occurring before MDD diagnosis or within 1 year after MDD diagnosis. Furthermore, the severity of depression at the first year of MDD diagnosis was recorded. Severe depression was determined based on the following criteria: 1) patient diagnosis matched ICD-9-CM codes: 296.23, 296.24, 296.33 or 296.34, or 2) the patient received an electroconvulsive therapy intervention during a depressive episode [30]. Prescription patterns during the first year following MDD onset and the information on medical care-seeking behavior were also evaluated. The average doses of antidepressants, lithium/mood stabilizers, and antipsychotics, as well as benzodiazepines and Z Drugs were determined. Early prescription pattern of combined treatment regimens (antidepressants and either antipsychotics or anticonvulsants) within 1 year of onset was analyzed.

### Statistical analysis

All analyses were performed using SAS, version 9.3. Differences between TRD and non-TRD groups were evaluated using the chi-squared test for categorical variables and the t-test for continuous variables. Bonferroni correction was used for multiple comparisons. Multiple Cox regression models were used to examine the effects of demographic variables and clinical features on the risk of developing TRD. The variables examined were sex, physical comorbidities, psychiatric comorbidities, diagnosis conversion, dose of antidepressants and benzodiazepines within 1 year since MDD onset, and the number of non-psychiatric outpatient visits 1 year before or after the onset of depression while adjusting for demographic covariates in the model (age, geographic area, urbanization level, and income estimated via insurance). Adjusted hazard ratios (aHRs) and 95% P for each covariate were reported for regression analyses. PAF for each significant comorbidity was assessed for the presence of TRD using Levin’s formula [31].

Survival analyses (from the onset of MDD to the occurrence of TRD) were performed to evaluate the distribution and proportion of TRD cases stratified by sex, comorbid FGIDs, comorbid thyroid dysfunction, severity of depressive symptoms within 1 year of onset, non-organic psychosis, and psychiatric comorbidities (anxiety disorders, SUDs, and personality disorders). Individuals who did not develop TRD, died, or dropped out were censored. A two-sided *p*-value of < 0.05 was considered statistically significant.

To evaluate the robustness of the study, sensitivity analysis was performed. In this analysis, early comorbidities were defined as only those occurring before the MDD onset. There were no significant differences between the different definitions.

## Results

The demographic and clinical characteristics of the patients are presented in Table 1. A total of 11,078 of 31,422 (35.26%) inpatients with MDD included in the study were defined as having TRD (21.24% of female and 14.02% of male patients). Females accounted for the majority of TRD patients than non-TRD patients (60.24% vs. 39.56%, *p* < 0.0001). The age of MDD onset was higher in the TRD group than in the non-TRD group (41.31 ± 15.33 vs. 37.85 ± 18.82, *p* < 0.0001). A greater percentage of patients in the TRD group had no income compared with that of those in the non-TRD group (21.82% vs. 16.3%, *p* < 0.0001). Patients with TRD exhibited a higher frequency of physical comorbidities, including diabetes, SLE, rheumatoid arthritis, cardiovascular disease, renal disease, FGIDs, and thyroid dysfunction, than non-TRD patients. All psychiatric comorbidities except ADHD were more prevalent in the TRD group than in the non-TRD group; these psychiatric comorbidities were personality disorders (24.34% vs. 15.07%, *p* < 0.0001), SUDs (33.03% vs. 16.64%, *p* < 0.0001), anxiety disorders (89.84% vs. 64.70%, *p* < 0.0001), panic disorder (15.63% vs. 6.02%, *p* < 0.0001), and non-organic psychosis (48.02% vs. 32.67%, *p* < 0.0001). During follow-up, a high proportion of patients with TRD had their diagnosis converted to BpD (22.53% vs. 10.57%, *p* < 0.0001) or schizophrenia (9.51% vs. 7.38%, *p* < 0.0001). Patients with TRD utilized medical care more frequently across all specialties than non-TRD patients. Overall medical care utilization was determined by calculating the number of hospital admissions plus ER visits (6.85 ± 15.17 vs. 2.40 ± 4.09, *p* < 0.0001) (Table 1). The average time from the onset of depression to TRD diagnosis was 3.32 years.

**Table 1:** Lifetime demographic and clinical characteristics between non-TRD and TRD patients

(*N* = 31,422)	non-TRD group (*N* = 20,344) 64.74%		TRD group (*N* = 11,078) 35.26%		*P* value^*^
Variable	N	%	N	%	
Female	8048	39.56	6673	60.24	< 0.0001
Urbanization level residence^a^					0.0014
I–III	16,188	79.61	8985	81.12	
IV–VII	4147	20.4	2092	18.88	
Estimated income via insurance (no income)	3262	16.3	2391	21.82	< 0.0001
All-cause mortality	2653	13.04	1464	13.22	0.6611
Lifetime physical comorbidity					
Diabetes mellitus	3183	15.65	2690	24.28	< 0.0001
Systemic lupus erythematosus	95	0.47	113	1.02	< 0.0001
Rheumatoid arthritis	308	1.51	356	3.21	< 0.0001
Cardiovascular disease	3607	17.73	2805	25.32	< 0.0001
Renal disease	1829	8.99	1330	12.01	< 0.0001
Functional gastrointestinal disorders	5250	25.81	4660	42.07	< 0.0001
Thyroid dysfunction	922	4.53	1058	9.55	< 0.0001
Lifetime psychiatric comorbidity					
Personality disorders	3065	15.07	2696	24.34	< 0.0001
Attention deficit hyperactivity disorder	103	0.51	57	0.51	0.9219
Substance use disorders	3385	16.64	3659	33.03	< 0.0001
Anxiety disorders	13,163	64.7	9952	89.84	< 0.0001
Panic disorder	1224	6.02	1732	15.63	< 0.0001
Non-organic psychosis	6646	32.67	5320	48.02	< 0.0001
Severe depression*	9338	45.90	6836	61.71	< 0.0001
Converted diagnosis to bipolar disorder	2150	10.57	2496	22.53	< 0.0001
Converted diagnosis to schizophrenia	1502	7.38	1053	9.51	< 0.0001
	Mean	SD	Mean	SD	
Age of onset for depression	37.85	18.82	41.31	15.33	< 0.0001
Age of onset for TRD	…	…	44.62	15.60	
Numbers of lifetime admissions for MDD	1.43	1.79	3.50	4.69	< 0.0001
Numbers of lifetime outpatient visits for MDD	18.68	31.28	73.29	71.56	< 0.0001
Overall medical care utilization for all subspecialties^b^	2.40	4.09	6.85	15.17	< 0.0001
Days from depression to bipolar disorder	1154.95	1080.22	1704.64	1125.91	< 0.0001
Days from depression to TRD	…	…	1211.53	1004.66	

*TRD* treatment-resistant depression, *MDD* major depression

Note ^a^ Seven levels, with one being the most urbanized and seven being the least urbanized

Note ^b^ Overall medical care utilization represented numbers of admissions and emergency room visits

Note ^*^ *P* value thresthold < 0.002 for Bonferroni correction

The early characteristics of patients with TRD are presented in Table 2. Generally, patients with TRD exhibited greater early physical comorbidities than patients without TRD. For early psychiatric comorbidities, patients with TRD had a higher prevalence of SUDs (15.68% vs. 10.58%, *p* < 0.0001), anxiety disorders (81.48% vs. 58.96%, *p* < 0.0001), and panic disorder (6.51% vs. 3.57%, *p* < 0.0001) than patients without TRD. However, patients with TRD had a lower prevalence of early personality disorders (8.34% vs. 10.64%, *p* < 0.0001), early non-organic psychosis (7.23% vs. 11.98%, *p* < 0.0001), and symptoms of severe depression within 1 year of onset (23.8% vs. 33.97%, *p* < 0.0001). A higher percentage of patients in the TRD group had their diagnosis converted to BpD (13.15% vs. 10.26%, *p* < 0.0001). Among all inpatients with MDD, 22.8% did not receive adequate treatment with antidepressants, antipsychotics, or anticonvulsants for 1 year since the onset of depression. During the first year of treatment for MDD, patients in the TRD group were prescribed significantly higher doses of antidepressants (279.90 vs. 142.90 DDD, *p* < 0.0001), lithium/mood stabilizers (DDD) (8.24 vs. 7.26 DDD, *p* < 0.0001), antipsychotics (26.63 vs. 23.79 DDD, *p* < 0.0001), as well as benzodiazepines and Z Drugs (528.70 vs. 260.41 DDD, *p* < 0.0001) than patients in the non-TRD group. Within 1 year of MDD onset, patients with TRD were also treated more frequently with combined regimens (antidepressants with either antipsychotics or anticonvulsants) than non-TRD patients (16.44% vs. 10.14%, *p* < 0.0001). Patients in the TRD group tended to have more outpatients visits than patients in the non-TRD group 1 year before or after the onset of MDD (psychiatric clinics, mean: 18.87 vs. 11.95, *p* < 0.0001; non-psychiatric clinics, mean: 50.09 vs. 42.31, *p* < 0.0001).

**Table 2:** Early comorbidities, prescription pattern, and behavior of seeking medical care between non-TRD and TRD patients

(*N* = 31,422)	non-TRD group (*N* = 20,344) 64.74%		TRD group (*N* = 11,078) 35.26%		*P* value^*^
Variable	N	%	N	%	
Early physical comorbidity^a^					
Diabetes mellitus	2182	10.73	1459	13.17	< 0.0001
Systemic lupus erythematosus	60	0.29	63	0.57	< 0.0001
Rheumatoid arthritis	178	0.87	135	1.22	0.0034
Cardiac vascular disease	2482	13.97	1943	17.54	< 0.0001
Renal disease	2689	13.22	1773	16	0.0018
Functional gastrointestinal disorders	3639	17.89	2682	24.21	< 0.0001
Thyroid dysfunction	556	2.73	515	4.65	< 0.0001
Early psychiatric comorbidity^a^					
Personality disorders	2164	10.64	924	8.34	< 0.0001
Attention deficit hyperactivity disorder	29	0.14	11	0.10	0.3043
Substance using disorders	2153	10.58	1737	15.68	< 0.0001
Anxiety disorders	11,994	58.96	9026	81.48	< 0.0001
Panic disorder	727	3.57	721	6.51	< 0.0001
Non-organic psychosis	2438	11.98	801	7.23	< 0.0001
Converted diagnosis to BpD within 1 year of onset	2087	10.26	1457	13.15	< 0.0001
Severe depression within 1 year of onset	6910	33.97	2637	23.8	< 0.0001
Prescription pattern within 1 year of onset					
Antidepressants plus antipsychotics or anticonvulsants	2062	10.14	1821	16.44	< 0.0001
	Mean	SD	Mean	SD	
Dosage of antidepressants (DDD)	142.90	152.90	279.90	223.40	< 0.0001
Dosage of lithium/mood stabilizers (DDD)	7.26	34.02	8.24	34.14	< 0.0001
Dosage of antipsychotics (DDD)	23.79	61.72	26.63	64.09	< 0.0001
Dosage of benzodiazepines and Z drugs (DDD)	260.41	324.74	528.70	529.58	< 0.0001
Health-seeking behavior for psychiatric medical care^b^					
Numbers of outpatient visits	11.95	9.75	18.87	13.78	< 0.0001
Health seeking behavior for non-psychiatric medical care^b^					
Numbers of outpatient visits	42.31	41.36	50.09	50.07	< 0.0001
Numbers of different physicians that patients visited	15.80	10.68	19.15	11.73	< 0.0001

*TRD* treatment-resistant depression, *BpD* bipolar disorder, *DDD* defined daily dose

Note^a^. Patients had comorbidity before depression onset or within 1 year after onset of depression

Note^b^. Duration was within 1 year prior or after onset of depression

Note^*^. *P* value threshold < 0.002 for Bonferroni correction

The results of the multiple Cox regression model are shown in Table 3. The model was adjusted for demographic features, including the age of onset, sex, geographic area, urbanization level, and income estimated by insurance. Significant variables were female gender, severe depression, lifetime psychiatric comorbidities (anxiety disorders, SUDs, personality disorders, and non-organic psychosis), lifetime physical comorbidities (diabetes, FGIDs, and thyroid dysfunction), conversion of the diagnosis to BpD, the daily dosage of antidepressants within 1 year of onset, and non-psychiatric outpatient visits 1 year before or after the onset of depression. Anxiety disorders exhibited the strongest effect associated with TRD (aHR = 2.02, 95% CI: 1.89–2.16), followed by the conversion of the diagnosis to BpD (aHR = 1.68, 95% CI: 1.61–1.75, SUDs (aHR = 1.38, 95% CI: 1.33–1.44), female sex (aHR = 1.24, 95% CI: 1.19–1.29), and personality disorders (aHR = 1.23, 95% CI: 1.17–1.29). The PAF of each comorbidity for TRD was calculated using aHR from the Cox regression model. The most common PAFs were lifetime anxiety disorders (42.87%), severe depression (10.59%), SUDs (7.85%), non-organic psychosis (6.75%), and FGIDs (5.65%). The average daily dose of antidepressants within 1 year since the onset of MDD (aHR = 1.002, 95% CI: 1.002–1.002, every DDD), the average daily doses of benzodiazepines and Z drugs (aHR = 1.002, 95% CI: 1.002–1.002, every 10 DDD), and the number of non-psychiatric outpatient visits 1 year before or after the onset of depression (aHR = 1.001, 95% CI: 1.000–1.001) were also significantly associated with an increased risk of TRD.

**Table 3:** Multivariable Cox regression^a^ analysis for risk of treatment-resistant depression

Variable	aHR	95% CI		PAF
Age of onset (depression) **	1.007	1.005	1.008	
Female**	1.24	1.19	1.29	
Lifetime physical comorbidities				
Diabetes mellitus*	1.09	1.04	1.14	1.65%
Cardiovascular disease	0.96	0.91	1.01	
Renal disease	0.96	0.90	1.02	
Functional gastrointestinal disorders**	1.19	1.15	1.24	5.65%
Thyroid dysfunction**	1.14	1.07	1.22	0.87%
Lifetime psychiatric comorbidities				
Personality disorders**	1.23	1.17	1.29	4.05%
Substance use disorders**	1.38	1.33	1.44	7.85%
Anxiety disorders**	2.02	1.89	2.16	42.87%
Non-organic psychosis**	1.19	1.14	1.24	6.75%
Severe depression**	1.23	1.17	1.29	10.59%
Converting diagnosis to bipolar disorder**	1.68	1.61	1.75	
Prescription pattern within 1 year of onset				
Dosage of antidepressants (defined daily dose)* *	1.002	1.002	1.002	
Dosage of benzodiazepine and its related drugs (every 10 defined daily dose)* *	1.002	1.002	1.002	
Numbers of non-psychiatric outpatient visits within 1 year prior or after onset of depression*	1.001	1.000	1.001	

*: *p*-value< 0.001;**: *p*-value< 0.0001

*CI* confidence Interval, *PAF* population attributable fraction, *aHR* adjusted hazard ratio

Note ^a^ Model adjusted for demographic features including geographic area, urbanization level, and income estimated via insurance

**Fig. 2:**
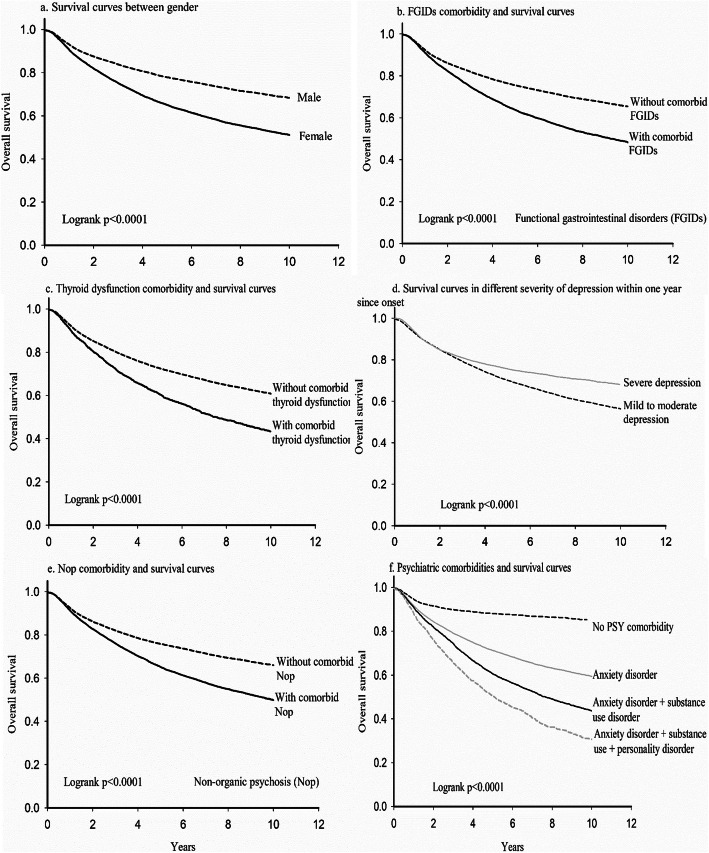
Survival curves of TRD in different models. 2a) for gender; 2b) for functional gastrointestinal disorders; 2c) for thyroid dysfunction; 2d) for severe depression within 1st year since onset; 2e) for nonorganic psychosis; 2f) for different psychiatric comorbidities

The results of the survival analyses are shown in Fig. 2. A significant difference in sex was identified between the groups (*p* < 0.0001). Physical comorbidities, including FGIDs and thyroid dysfunction, were associated with a higher frequency of TRD diagnosis during follow-up (*p* < 0.0001). Surprisingly, patients with early severe depressive symptoms had better prognosis than those without early severe symptoms (*p* < 0.0001). The survival rate was significantly reduced in patients with more than one comorbid psychiatric condition. The 10-year survival rate was approximately 31% in patients with multiple psychiatric comorbidities (anxiety disorders, SUDs, and personality disorders) compared with those with no prior psychiatric comorbidities (85%).

## Discussion

Using a nationwide database in the current study, one of three MDD patients have TRD. Furthermore, our results showed that patients with multiple psychiatric comorbidities have a lower ‘survival rate’ (not developing TRD) than those without these conditions (31% vs. 85%). The large registry-based claims data are generally representative as the registration rate is high (97%) and all data regarding prescribed medications are documented. This type of study is particularly beneficial when long-term clinical management is recorded, including the duration of depressive episodes within a pre-specified patient population and clinical characteristics (e.g., comorbid conditions) [6, 32]. However, claims data may not include the assessment of subjective treatment response and treatment adherence, and a poor response to medication may be underestimated. The definition of TRD in the current study was adapted from a previous study of claims data and was determined based on whether the patient’s antidepressant treatment regimen was altered two or more times [5–7]. However, we cannot dismiss the possibility that some patients who had continued taking medication still experienced severe depression. To complement the criteria of altering medication, this definition was further added: if the patient received two or more adequate antidepressant treatment regimens but was subsequently admitted in a psychiatric acute ward. This amended definition may represent a useful proxy to capture treatment failure and allow for better identification of TRD in patients who remain in clinical settings [7, 33, 34].

The current study identified the female sex as a risk factor of TRD (aHR = 1.24). A cross-sectional study in the UK that used questionnaires to collect treatment history found that 70% of patients with TRD were females [34], which is consistent with our findings. Furthermore, increased vulnerability to depression in women begins at puberty and declines after menopause [35]. One possible explanation is that the hypothalamic–pituitary–adrenal function (cortisol levels) is more likely to fluctuate in response to stressors and during depressive episodes in women [36] . Redox imbalance elicited by estrogen has been revealed to weaken enzymatic antioxidant defenses, which may be linked to TRD [37, 38]. Although several physical comorbidities increase the risk of depression [39], it is unknown whether these comorbidities increase the risk of TRD. The current study illustrated that patients with TRD had more physical comorbidities than those without TRD during long-term follow-up, including diabetes, FGIDs, and thyroid dysfunction. Growing evidence suggests a bidirectional communication between the gastrointestinal tract and brain [40]. An unhealthy gastrointestinal tract may result in treatment complications and reduce responsiveness to antidepressant treatment. Other studies have found that antidepressants may be used to treat FGIDs [41]. Previous studies have also indicated that thyroid dysfunction, which is more prevalent in women, also influences treatment outcomes [37, 42]. The aHR of these physical comorbidities ranged from 1.09 (diabetes mellitus) to 1.19 (FGIDs). Taken together, these risk factors have a mild yet significant impact on the development of TRD and required further study.

We reported that long-term psychiatric comorbidities are important and independent risk factors of TRD development. In particular, anxiety disorders were observed in > 80% of MDD patients in this nationwide database. We reported a 2.02-fold higher risk of TRD development in patients with comorbid anxiety disorders. In addition, our results revealed that anxiety is the strongest predictor of TRD with a high PAF. It is well-known that anxiety and depression/TRD often coexist [22, 23, 25], and that anxiety disorders have been identified as a risk factor of poor treatment response [43]. A previous study also found that anxiety disorders, particularly panic disorder, are associated with TRD [44]. There is evidence that depression and anxiety disorders have additive effects on increased disability and poorer recovery from medical illness [45]. Patients with comorbid depression and anxiety have more severe somatic symptoms than those MDD patients without anxiety [46], which may predispose them to sensitive to side effects from medication and dropping out of treatment prematurely [47]. In addition, comorbid anxiety in depressed patients is associated with poorer social function [45], which may correlate with poor social support. Besides, depression with comorbid symptoms of anxiety is associated with greater suicidal ideation [45]. Possibly as a result of increased illness severity, depressed patients are more likely to be prescribed benzodiazepines if they have anxiety symptoms [48]. It was also consistent with our finding of high usage of benzodiazepines use, because these drugs were prescribed two-fold more often in the TRD group than in the non-TRD group. This suggests that even in the early phase of MDD, comorbid anxiety disorders represent a red-flag in clinical settings that increases the difficulty of patient care and management and increases the risk of TRD development.

Except anxiety disorders, there are several psychiatric comorbidities correlated with TRD. A previous study found that certain personality traits, such as low cooperativeness and high neuroticism, positively correlate with TRD [13]. Personality disorders correlate with poor drug adherence, poor social function, and more SUDs, which may further result in TRD [49]. Our findings are in line with the observation that personality disorders are associated with a 1.23-fold higher risk of TRD. In addition, SUDs represent another important comorbidity condition of MDD [50]. One review article has found that mood disorders are common in patients with SUDs, whereas in many individuals, the mood disturbance cannot be attributed to the acute effects of substance use or withdrawal [51]. Mood disorders may trigger individuals to use drugs and alcohol to cope with negative affective states. Substance use is often viewed as a self-medication method to alleviate depression or anxiety. Meanwhile, substance use may mask underlying mood disturbances and aggravate symptoms, ultimately leading to TRD [52]. SUDs are also risk factors of several psychiatric disorders, including anxiety disorders; therefore, the effect on the increased risk of TRD may be confounded by SUDs. After adjustment in multiple Cox regression models, we reported that several psychiatric disorders remained independent risk factors.

The relationships between comorbidities and TRD have been examined in a cross-sectional manner without clear temporal consequences in the literature [6, 12, 13]. In the present study, using Cox regression models and plots with survival function, our results demonstrated the temporal relationship that early and long-term psychiatric comorbidities significantly increase the risk of subsequent treatment resistance events. The aHRs of psychiatric comorbidities ranged from 1.19 to 2.02; the point estimates are generally higher than those of physical comorbidities. Furthermore, approximately 70% of patients with multiple lifetime psychiatric comorbidities developed TRD in this study. On the other hand, non-organic psychosis, and severe depression at the first year of diagnosis reduced the risk of TRD. It is possible that patients with initial severe and psychotic depression may have been treated more thoroughly or prescribed with a higher dose of antidepressant medication. Taken together, the comorbidity profiles of TRD are important and warrant further evaluation for the early detection of TRD.

In the current study, the patients in the TRD group visited non-psychiatric clinics more often than those in the non-TRD group. Patients in the TRD group more commonly visited non-psychiatric clinics than psychiatric clinics during their first year of treatment. Interestingly, 65% of patients with depression sought help at general medical clinics prior to a psychiatric clinic [53]. Another clinical study reported that somatic symptoms prevailed in a great majority of depressed patients; these patients may initially seek non-psychiatric medical care before psychiatric care [54]. Patients with depression may delay proper psychiatric treatment, resulting in an increased risk of treatment resistance. Therefore, it is important to evaluate patients with depression who have frequently visit non-psychiatric clinics and provide them suitable treatment. Furthermore, both health-seeking behavior and drug compliance may be correlated with treatment resistance. Adherence was found to play an important role in TRD development and increased the risk of mortality [8, 14, 26]. In the claims database, adherence was not easy to assess. On the other hand, patients with very poor drug adherence were unlikely to fulfill the definition of TRD for having a number of antidepressant trials. Our analysis revealed no significant difference in the mortality rate between the TRD and non-TRD groups, possibly due to the relatively young age of the patients and relatively short follow-up period in the database.

We also found that patients in the TRD group had significantly higher doses of psychotropic prescriptions and a greater incidence of long-term use than those in the non-TRD group within 1 year of MDD diagnosis. The antidepressants dose remained significantly associated with TRD in multiple analysis. Long-term treatment with antidepressants might have contributed to the subsequent treatment resistance [55]. There are some possible explanations for this phenomenon. First, treating patients with mood disorders using antidepressants may have a paradoxical effect (exacerbate depression) [56, 57]. Second, previous studies have described antidepressant-induced switching and cycle acceleration in patients with BpD [58]. In the current study, patients whose diagnosis was converted to BpD experienced a 1.72-fold higher risk of developing TRD. Third, tolerance to antidepressants has been reported [59]. Patients with TRD also had more complex treatment regimens, because it is a common strategy to use higher doses and adjunctive psychotropics to counter poor response to medication [60]. A recent meta-analysis of placebo-controlled trials demonstrated that adjunctive antipsychotics were effective for the treatment of TRD [61]. Another meta-analysis demonstrated the efficacy of lithium augmentation with antidepressants compared with the placebo for the treatment of TRD [62]. However, because the current study was not a randomized, placebo-controlled trial, we did not compare a combined treatment regimen with monotherapy. Rather, we found that patients eventually developing TRD required early attention and more complex treatment at an early disease stage. The results of the current study suggest that early prescription patterns in patients represents a proxy for the combined treatment regimen and poor response/prognosis.

Current treatment regimens for depression sometimes have limited efficacy. Novel antidepressants with different mechanisms are currently used as treatment options for TRD, including agents targeting the hypothalamic–pituitary–adrenal axis, glutamate/GABA system, or opioid receptors [63]. One promising agent is ketamine, which is a noncompetitive NMDA receptor antagonist that can produce rapid and stable antidepressant effects [64]. Several ongoing clinical trials of ketamine for the treatment of TRD are ongoing in Taiwan; they have revealed satisfactory effects [65, 66]. Other widely discussed antidepressant agent are cannabinoid compounds because the endocannabinoid system is widely distributed throughout the body and serves to modulate mood symptoms [67].

This study has several limitations that need to be considered when interpreting the results. First, structured interview data were not available because we solely relied on claims data. Furthermore, more detailed clinical features and the assessments of disease severity were not available. Second, we could not obtain information on uninsured subjects. However, this study is highly representative of the target population because approximately 97% of the population was insured. Third, we targeted patients who had ever been admitted to a hospital because of depressive episodes. The results may not be extrapolated to all MDD patients, particularly to those who had never been admitted. Fourth, it was not possible to evaluate patients’ true treatment compliance. Nevertheless, we attempted to exclude those with poor compliance (e.g., patients filling prescriptions for less than 56 days). Fifth, we intended to investigate the early features of the TRD group; however, we did not have information on the subjects before they sought medical help. Sixth, we did not consider the patient’s lifestyle and personal history (i.e., coping strategies, exercise habits, smoking, psychological trauma, etc.), which might have influenced the incidence of TRD. Finally, the mortality rate may be underestimated because death records were missing from the dataset and we cannot clarify the primary causes of death. Therefore, future studies are warranted to link this database with national death records to answer such questions.

## Conclusions

At least one of three ever admitted patients with MDD developed TRD in this study. These patients visited non-psychiatric medical care more frequently than specialized psychiatric facilities and were prescribed higher doses of psychotropic agents within the first year of MDD diagnosis. In addition, patients with TRD had lower incomes and utilized medical services more frequently than those without TRD. Female patients exhibited a higher risk of TRD than male patients. Our findings indicated that physical and psychiatric comorbidities substantially increase the risk of TRD during follow-up, and that 70% of patients with multiple psychiatric comorbidities developed TRD during follow-up. Clinicians should be more aware of patients’ physical and psychiatric comorbidity patterns and early polypharmacy to better develop comprehensive treatment plans and minimize the risk of developing TRD.

## Data Availability

The datasets generated and/or analyzed of the current study are not publicly available due to the privacy policy of statistics department of Ministry of Health and Welfare, Taiwan.

## Abbreviations

TRD: Treatment-resistant depression
MDD: Major depressive disorder
ER: Emergency room
FGIDs: Functional gastrointestinal disorders
SLE: Systemic lupus erythematosus
ADHD: Attention deficit hyperactivity disorder
SUDs: Substance use disorders
PAF: Population attributable fraction
PIMC: Psychiatric Inpatients Medical Claim
BpD: Bipolar disorder
DDD: Defined daily doses
aHRs: Adjusted hazard ratios
CI: Confidence interval
